# Fourteen-day vonoprazan and low- or high-dose amoxicillin dual therapy for eradicating *Helicobacter pylori* infection: A prospective, open-labeled, randomized non-inferiority clinical study

**DOI:** 10.3389/fimmu.2022.1049908

**Published:** 2023-01-13

**Authors:** Yi Hu, Xin Xu, Xiao-Shun Liu, Cong He, Yao-Bin Ouyang, Nian-Shuang Li, Chuan Xie, Chao Peng, Zhen-Hua Zhu, Yong Xie, Xu Shu, Yin Zhu, David Y. Graham, Nong-Hua Lu

**Affiliations:** ^1^ Department Of Gastroenterology, Digestive Disease Hospital, The First Affiliated Hospital of Nanchang University, Nanchang, Jiangxi, China; ^2^ JiangXi Clinical Research Center for Gastroenterology, Nanchang, Jiangxi, China; ^3^ Department of Medicine, Michael E. DeBakey VA Medical Center, and Baylor College of Medicine, Houston, TX, United States

**Keywords:** *Helicobacter pylori*, vonoprazan, amoxicillin, dual therapy, efficacy

## Abstract

**Background and aim:**

We previously reported that vonoprazan-amoxicillin (VA) dual therapy for 7 or 10 days is not satisfactorily efficacious for *Helicobacter pylori* (*H. pylori*) eradication. We aimed to explore the efficacy of VA dual therapy for 14 days as a first-line treatment for *H. pylori* infection.

**Methods:**

This was a single center, prospective, open-labeled, randomized non-inferiority clinical study conducted in China. Treatment naïve *H. pylori* infected patients were randomized into two groups: 20 mg vonoprazan (VPZ) b.i.d. in combination with low-dose (1000 mg b.i.d.) or high-dose (1000 mg t.i.d) amoxicillin for 14 days. ^13^C-urea breath tests were used to access the cure rate at least 4 weeks after treatment.

**Results:**

A total of 154 patients were assessed and 110 subjects were randomized. The eradication rate of VPZ with b.i.d. amoxicillin or t.i.d. amoxicillin for 14 days was 89.1% and 87.3% by intention-to-treat analysis, respectively, and 94.1% and 95.9% by per-protocol analysis, respectively. The eradication rate and incidence of adverse events were not different between the two groups.

**Conclusion:**

VPZ with b.i.d. or t.i.d. amoxicillin for 14 days provides satisfactory efficacy as a first-line treatment for *H. pylori* infection in China.

## Introduction

*Helicobacter pylori* (*H. pylori*) infection is considered the primary etiological and the most important risk factor for gastric cancer (GC) (1). This has resulted in worldwide efforts to eradicate the infection. Additionally, Family-based *H. pylori* infection control and management has been recommended as an essential part of comprehensive *H. pylori* infection prevention and control strategies (2). China has a high incidence of GC (3) and a high prevalence of *H. pylori* infection (4), and *H. pylori* eradication has been recommended by the Fifth Chinese National Consensus Report on the management of *H. pylori* infection (5). Considering the serious situation of antibiotic resistance in China, bismuth-containing quadruple therapy has been recommended as the first-line treatment of *H. pylori* because of its high efficacy and safety (5).

Vonoprazan (VPZ), a new potassium-competitive acid blocker, has been shown to be a more potent inhibitor of gastric acid and is able to maintain the intragastric pH above 6 more quickly and for longer than traditional proton pump inhibitors (PPI) (6). Maintaining gastric pH above 6 allows *H. pylori* to remain in the state of active replication, which enhances the effectiveness of amoxicillin. We previously conducted a systematic review and meta-analysis of three Japanese studies with 668 *H. pylori*-infected patients designed to evaluate the efficacy of vonoprazan-amoxicillin (VA) dual therapy as a first-line treatment of *H. pylori* infection. The crude eradication rate of VA dual therapy was 87.5% by intention-to-treat (ITT) analysis and 89.6% by per-protocol (PP) analysis (7). VA dual therapy is considered as a promising *H. pylori* regimen because of its high efficacy, low side effects, and avoidance of unnecessary antibiotic use (8). However, the efficacy and safety of VA dual therapy in other regions remain uncertain, thus requiring the regimen to be optimized.

Our previous study included 119 *H. pylori-*infected Chinese patients without previous eradication history who were randomized into low- or high-dose amoxicillin-vonoprazan regimen groups consisting of 1 g amoxicillin either b.i.d. or t.i.d plus 20 mg VPZ b.i.d for 7 or 10 days. Neither 7- or 10-day VA dual therapy with either b.i.d. or t.i.d. amoxicillin achieved satisfactory efficacy (i.e., <90%) when administered as the first-line treatment of *H. pylori* infection (9). This study evaluated the efficacy and safety of 14-day low- and high-dose amoxicillin-vonoprazan dual therapy as a first-line treatment of *H. pylori* in China.

## Methods

### Study design

This study was designed as a prospective open-labeled randomized non-inferiority clinical study and was conducted in accordance with the Declaration of Helsinki and the guidelines of the Consolidated Standards of Reporting Trials (10). This study was approved by the Ethics Committee of The First Affiliated Hospital of Nanchang University (2022-013) and registered in the Chinese Clinical Trial Registry (ChiCTR2000064874).

### Study population

Consecutive *H. pylori-*infected subjects aged from 18 to 70 years old without eradication history were recruited in The First Affiliated Hospital of Nanchang University between November 2021 to February 2022. *H. pylori* infection was confirmed by immunohistochemistry or ^13^C-urea breath test. Patients were excluded if they met any of the following criteria (1): allergy to amoxicillin or VPZ (2); acute upper gastrointestinal bleeding, GC or other tumors, Zollinger–Ellison syndrome, history of gastric surgery (3); serious illness, including neurological, cardiovascular, pulmonary, hepatic, renal, metabolic, gastrointestinal, urological, endocrinological, or hematological disorders (4); pregnancy or breastfeeding (5); proton pump inhibitor and antibiotic use within 1 month (6); not willing to participate in the study. Written informed consent was obtained from all patients prior to study participation.

### Randomization and treatment

The 110 *H. pylori-*infected subjects were randomly assigned to receive either low- or high-dose amoxicillin-vonoprazan dual therapy in a 1:1 allocation ratio (a randomization list was generated using SPSS [version 25.0]). Low-dose amoxicillin-vonoprazan (L-VA) dual therapy consisted of 1000 mg amoxicillin capsules (Huabei Laboratories, Shijiazhuang, China) twice daily and 20 mg VPZ fumarate tablets (Takeda Pharmaceutical, Tokyo, Japan) twice daily for 14 days. High-dose amoxicillin-vonoprazan (H-VA) dual therapy consisted of 1000 mg amoxicillin capsules (HuaBei Laboratories, Shijiazhuang, China) three times daily and 20mg VPZ fumarate tablets (Takeda Pharmaceutical, Tokyo, Japan) twice daily for 14 days. Patients and investigators were not blinded to the allocated treatment group.

### Procedures

To begin with, the detailed demographics and characteristics of the subjects included in this study were recorded, including sex, age, nationality, height, weight, education status, dwelling area, history of smoking and alcohol, concomitant diseases, and medication history. In addition, physical examinations and assessments of vital signs were performed.

During (or after) treatment-emergent adverse events (TEAEs) and concomitant medication were recorded throughout the study, including bloating, nausea, vomiting, abdominal pain, diarrhea, constipation, skin rash, headache, and hunger sensation. All TEAEs were divided into mild, moderate, and severe. TEAEs leading to the discontinuation of the study drug were also recorded. The confirmation of *H. pylori* status was evaluated by ^13^C-urea breath test at least 4 weeks after treatment. *H. pylori* status was considered as negative or positive when the delta over baseline was below 4 or above 4 according to the manufacturer’s instructions (HCBT-01, Shenzhen Zhonghe Headway Bio-Sci & Tech Co., Ltd., China).

The primary outcome of the study was the eradication rate of the different regimens according to ITT and PP analyses. The ITT analysis included all randomized patients. Patients who were lost to follow-up or did not achieve >80% of drug compliance or did not undergo UBT were excluded from the PP analysis. The secondary outcomes were the frequency and severity of TEAEs and adherence.

### Sample size calculation and statistical analysis

The sample size was calculated using PASS (version 11.0.7) according to non-inferiority for two proportions. In our previous study, the eradication rate of L-VA and H-VA dual therapies for 10 days was 89.2% and 81.1%, respectively, as a first-line treatment of *H. pylori* infection (9), which suggested that low-dose amoxicillin-vonoprazan dual therapy might be non-inferior to high dose. We assumed that VA dual therapy for 14 days would achieve a higher efficacy than that for 10 days. Moreover, acceptable therapy was defined as achieving >90% cure rates in adherent patients with susceptible infection (11). The eradication rate of L-VA and H-VA dual therapies for 14 days was estimated as 90%. Assuming a power, an alpha, a non-inferiority margin, and a follow-up loss rate of 0.80, 0.05, −0.15, and 10%, respectively, were required for a non-inferiority clinical trial, a sample size of 110 patients (55 patients in each group) was planned.

The primary outcome was calculated for each regimen *via* ITT and PP analyses. The differences in eradication rate were compared using the chi-square test. The quantitative data with normal or non-normal distribution were analyzed by Student’s t-test and a non-parametric test, respectively. The categorical data among different regimens were compared using the chi-square test. All statistical analyses were performed using SPSS (version 25.0) and R (version 4.2.1). All P-values were two-sided, except for the test of non-inferiority. P<0.05 was considered statistically significant.

## Results

### Baseline characteristics

**Figure 1:**
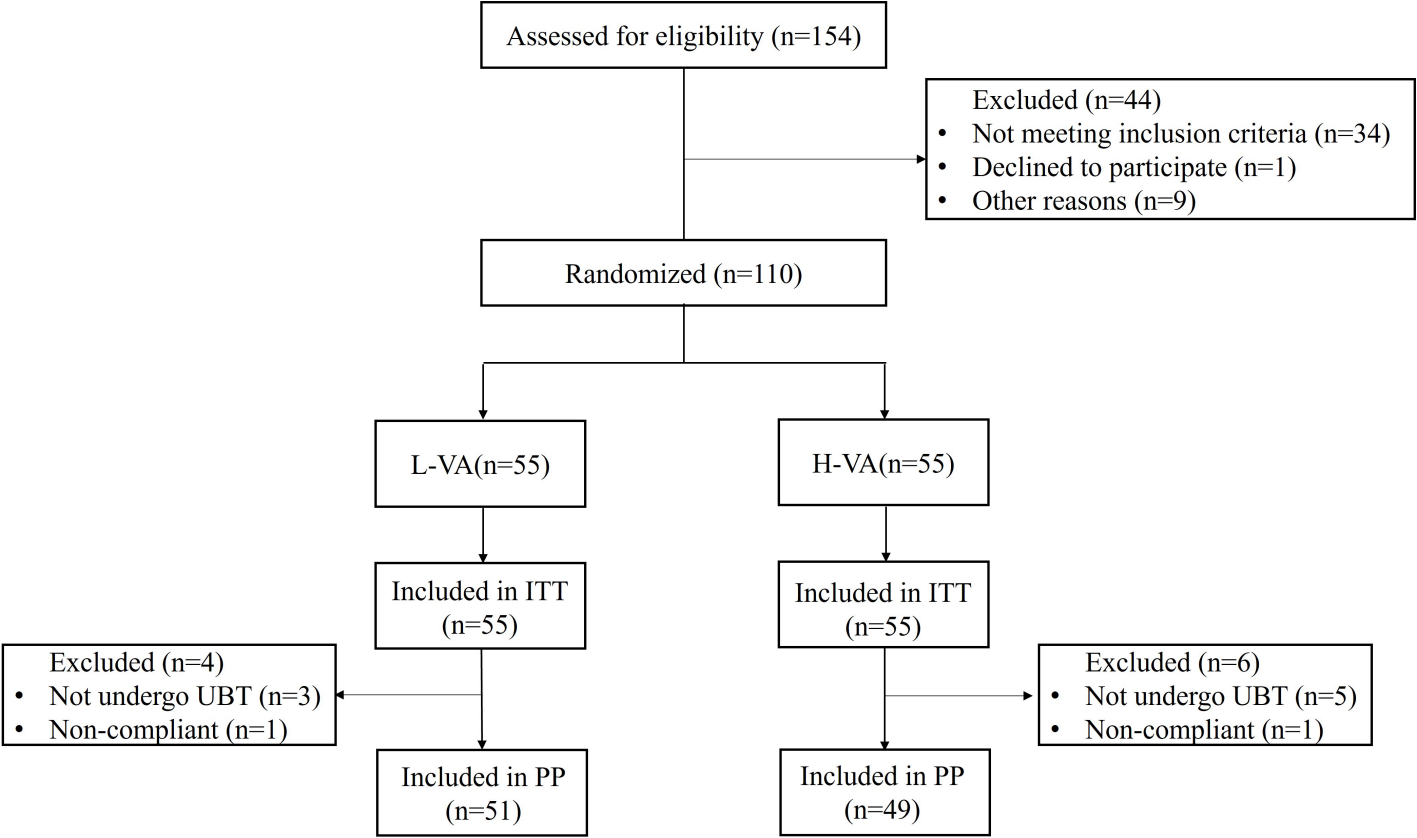
Flow chart of patient enrollment. ITT, intention to treat; PP, per protocol; L-VA, dual therapy consisting of low-dose amoxicillin (1000 mg b.i.d.) and vonoprazan (20 mg b.i.d.); H-VA, dual therapy consisting of high-dose amoxicillin (1000 mg t.i.d.) and vonoprazan (20 mg b.i.d.).

A total of 154 participants were assessed for eligibility between November 2021 and February 2022. Among these, 44 patients were excluded because they did not meet the inclusion criteria (n=34), declined to participate (n=1), or for other reasons (n=9). Finally, 110 patients were enrolled and randomized into two groups that received either L-VA or H-VA dual therapy. Final follow-up was completed on the 31st of August 2022. One patient in each group was non-compliant, and three patients in the L-VA group and five patients in the H-VA group did not take a ^13^C-urea breath test (**Figure 1**). Baseline characteristics (age, sex, body mass index, education status, cigarette smoking, alcohol drinking, and indications) showed no major differences between groups (**Table 1**).

**Table 1:** Baseline characteristics of the participants.

Characteristic	L-VA (n=55)	H-VA (n=55)	*P* value
Age (years, mean±SD)	41.1±12.1	40.1±13.1	0.7
Range	20-69	20-68	
Sex (male/female)	22/33	27/28	0.337
BMI (kg/m^2^, mean±SD)	22.9±3.4	22.0±4.0	0.211
Range	16.9-32.2	16.4-33.1	
Education status			0.848
High school or less	24 (43.6%)	25 (45.5%)	
College or more	31 (56.4%)	30 (54.5%)	
Cigarette smoking	8 (14.5%)	10 (18.2%)	0.606
Alcohol drinking	18 (32.7%)	18 (32.7%)	1
Indications			
Peptic ulcers	6 (10.9%)	5 (9.1%)	0.466
Dyspepsia	2 (3.6%)	6 (10.9%)	
Gastritis	20 (36.4%)	16 (29.1%)	
*H. pylori* confirmed	27 (49.1%)	28 (50.9%)	

BMI, body mass index; L-VA, dual therapy consisting of low-dose amoxicillin (1000 mg b.i.d.) and vonoprazan (20 mg b.i.d.); H-VA, Dual therapy consisting of high-dose amoxicillin (1000 mg t.i.d.) and vonoprazan (20 mg b.i.d.).

### Efficacy

The *H. pylori* eradication rates for L-VA or H-VA dual therapy are shown in **Table 2**. In the ITT analysis, the *H. pylori* eradication rate was 89.1% (49/55, 95% CI 77.1% to 95.5%) for L-VA dual therapy and 87.3% (48/55, 95% CI 74.9% to 94.3%) for H-VA dual therapy, with the difference between each group being 1.8% (95% CI −12.1% to 15.7%). In the PP analysis, the *H. pylori* eradication rate was 94.1% (48/51, 95% CI 82.8% to 98.5%) for L-VA dual therapy and 95.9% (47/49, 95% CI 84.9% to 99.3%) for H-VA dual therapy, with the difference between each group being 1.8% (95% CI −10.1% to 13.6%).

**Table 2:** Eradication rate of each treatment group.

Analysis	L-VA	H-VA	Difference from H-VA (adjusted 95% CI for difference)	*P* value for difference*	*P* value for non-inferiority †
ITT	89.1% (49/55)	87.3% (48/55)	1.8% (-12.1% to 15.7%)	0.768	0.616
95% CI	77.1% to 95.5%	74.9% to 94.3%			
PP	94.1% (48/51)	95.9% (47/49)	1.8% (-10.1% to 13.6%)	0.68	0.339
95% CI	82.8% to 98.5%	84.9% to 99.3%			

*P-values were obtained from two-sided comparisons of the difference between the L-VA group and the H-VA group; †The P values were obtained from one-sided test comparisons of non-inferiority between the L-VA group and the H-VA group. ITT, intention to treat; PP, per protocol; L-VA, dual therapy consisting of low-dose amoxicillin (1000 mg b.i.d.) and vonoprazan (20 mg b.i.d.); H-VA, dual therapy consisting of high-dose amoxicillin (1000 mg t.i.d.) and vonoprazan (20 mg b.i.d.).

The lower bound of the adjusted 95% CI for the efficacy difference between L-VA dual therapy and H-VA dual therapy was greater than the prespecified non-inferiority margin in the ITT and PP analyses. No differences in the eradication rate between the two groups were observed according to ITT and PP analysis.

### Treatment-emergent adverse events and compliance

TEAEs occurred in 16 patients in the L-VA dual therapy group and in 11 patients in the H-VA dual therapy group. One patient in the L-VA group presented moderate TEAEs. No patients discontinued the administration of study drugs because of TEAEs. The incidence of TEAEs between the two groups was not statistically different (29.1% vs. 20.0%, P=0.268) (**Table 3**). In the L-VA or H-VA dual therapy groups, 98.2% of patients took at least 80% of the study drugs. No factors were associated with the efficacy of VA dual therapy in the per-protocol population (**Table 4**).

**Table 3:** Treatment-emergent adverse events and patient compliance of each treatment group (n, %).

Characteristic	L-VA	H-VA	P-value
Bloating	2 (3.6%)	2 (3.6%)	
Nausea/vomiting	1 (1.8%)	2 (3.6%)	
Abdominal pain	4 (7.3%)	0 (0%)	
Diarrhea	2 (3.6%)	2 (3.6%)	
Constipation	1 (1.8%)	0 (0%)	
Skin rash	2 (3.6%)	2 (3.6%)	
Headache	1 (1.8%)	0 (0%)	
Moderate	1 (1.8%)	0 (0%)	
Hunger sensation	1 (1.8%)	1 (1.8%)	
Hiccup	0 (0%)	1 (1.8%)	
Acid reflux	1 (1.8%)	0 (0%)	
Bitter taste	1 (1.8%)	1 (1.8%)	
Total	16 (29.1%)	11 (20.0%)	0.268
Mild	15 (27.3%)	11 (20.0%)	0.369
Moderate	1 (1.8%)	0 (0%)	0.315
Severe	0 (0%)	0 (0%)	1.000
Leading to study drug discontinuation	0 (0%)	0 (0%)	1.000
Compliance	54 (98.2%)	54 (98.2%)	1.000

L-VA, dual therapy consisting of low-dose amoxicillin (1000 mg b.i.d.) and vonoprazan (20 mg b.i.d.); H-VA, dual therapy consisting of high-dose amoxicillin (1000 mg t.i.d.) and vonoprazan (20 mg b.i.d.).

**Table 4:** Factors influencing cure rates in the per-protocol population.

Variables	L-VA	H-VA
Gender		
Male	19/21 (90.5%)	23/25 (92.0%)
Female	29/30 (96.7%)	24/24 (100.0%)
*P* value	0.355	0.157
Age		
<45	25/26 (96.2%)	30/31 (96.8%)
≥45	23/25 (92.0%)	17/18 (94.4%)
*P* value	0.529	0.691
BMI		
<24	30/32 (93.8%)	33/35 (94.3%)
≥24	18/19 (94.7%)	14/14 (100.0%)
*P* value	0.885	0.361
Education status		
High school or less	22/23 (95.7%)	23/24 (95.8%)
College or more	26/28 (92.9%)	24/25 (96.0%)
*P* value	0.673	0.976
Cigarette smoking		
Yes	7/7 (100.0%)	9/10 (90.0%)
No	41/44 (93.2%)	38/39 (97.4%)
*P* value	0.476	0.289
Alcohol drinking		
Yes	17/18 (94.4%)	17/17 (100.0%)
No	31/33 (93.9%)	30/32 (93.8%)
*P* value	0.942	0.293
Family-based *H. pylori* infection		
Yes	19/20 (95%)	11/11 (100.0%)
No	29/31 (93.5%)	36/38 (94.7%)
*P* value	0.83	0.437
Indications		
Peptic ulcers	6/6 (100.0%)	4/4 (100.0%)
Dyspepsia	2/2 (100.0%)	4/5 (80.0%)
Gastritis	15/18 (83.3%)	14/14 (100.0%)
*H. pylori* confirmed	25/25 (100.0%)	25/26 (96.2%)
*P* value	0.119	0.261

BMI, body mass index; L-VA, dual therapy consisting of low-dose amoxicillin (1000 mg b.i.d.) and vonoprazan (20 mg b.i.d.); H-VA, dual therapy consisting of high-dose amoxicillin (1000 mg t.i.d.) and vonoprazan (20 mg b.i.d.).

## Discussion

To the best of our knowledge, this is the first randomized clinical trial to investigate the efficacy of VPZ in combination with low- or high-dose amoxicillin for 14 days as a first-line treatment of *H. pylori* infection. We found that the efficacy of L-VA (PP analysis: 94.1%) was non-inferior to H-VA (PP analysis: 95.9%) for eradicating *H. pylori*. L-VA and H-VA dual therapies for 14 days were defined as at least conditionally acceptable according to the clinical definitions of outcome^11^. Moreover, low side effects, good compliance, and safety were achieved by VA dual therapy.

VPZ is a member of a new class of acid-suppressing agents that inhibit the enzyme by reversible K^+^-competitive ionic binding and do not require acid activation within the parietal cell secretory canaliculus. VPZ has been widely applied to treat acid-related diseases, including *H. pylori* infection (12). The 24-h pH>4 and pH>5 holding time ratios were 83.4% and 73.2%, respectively, in a Japanese population when 20 mg VPZ was administered for 7 consecutive days. The holding time ratio increased to 100% and 98.6%, respectively, when 40 mg VPZ was administered once daily (13). However, the inhibition ability of gastric acid induced by VPZ was slightly decreased in the UK compared with that in Japan, which might be due to the difference in body mass index between the two regions. VPZ has been used at a dose of 20 mg twice daily in *H. pylori* regimens. The 24-h pH>4 and pH>5 holding time ratios were 68% and 54%, respectively, when 20 mg esomeprazole was administered twice daily, which is lower than that achieved with 20 mg VPZ b.i.d (6).

The primary and secondary resistance rates to clarithromycin are now above alarming levels (>15%) worldwide (14), and clarithromycin-resistant *H. pylori* was included in a high priority list of antibiotic-resistant bacteria that needed effective drugs (15). Approximately 80% of infected patients received no additive benefits from clarithromycin use in VPZ triple therapy, which has also contributed to global antimicrobial resistance (16). Suzuki et al. (17) produced one of the first studies that explored the efficacy of VPZ (20 mg b.i.d.) with low-dose amoxicillin (750 mg b.i.d.) for 7 days as a first-line treatment of *H. pylori* infection in Japan. VA dual therapy achieved 84.5% in ITT analysis and 87.1% in PP analysis, which was non-inferior to VPZ-based triple therapy in the same population. A similar efficacy (ITT analysis, 85.0%; PP analysis, 86.4%) was also achieved by VA dual therapy (20 mg VPZ and 750 mg amoxicillin twice daily) for 7 days in junior high school students in Japan (18). Eto et al. (19) further analyzed the factors that might influence the efficacy of VA dual therapy. A total of 163 *H. pylori*-infected subjects were included and the total eradication rate was 87.1%. High body surface area or body mass index have been associated with the decreased efficacy of VA dual therapy. The plasma half-life of amoxicillin is short, and an increase in the frequency of amoxicillin administered daily will theoretically improve the efficacy of amoxicillin. A retrospective study (20) was conducted in Japan to evaluate the efficacy of VPZ (20 mg b.i.d.) in combination with amoxicillin (500 mg t.i.d.) for 7 days as a first-line treatment of *H. pylori* infection. According to ITT analysis, VA dual therapy achieved an eradication rate of 92.9%, which was non-inferior to VPZ-based triple therapy.

China has a high resistance rate to antibiotics (21). However, the resistance rate to amoxicillin has remained relatively low (21), especially in Jiangxi province (22). VPZ was introduced in China in 2019 and the efficacy of VA dual therapy for eradicating *H. pylori* in China remained unclear. In our previous study, 119 *H. pylori*-infected patients without *H. pylori* eradication history were randomized into two groups: 20 mg VPZ b.i.d. with low- (1000 mg b.i.d.) or high-dose (1000 mg t.i.d.) amoxicillin for 7 or 10 days. We found that VPZ with low-or high-dose amoxicillin achieved unsatisfactory efficacy when administered for 7 or 10 days (9), suggesting that a longer duration was needed. We designed a prospective open-labeled randomized non-inferiority clinical study because VPZ in combination with low-dose amoxicillin for 10 days showed a higher eradication rate than that with a high dose, although the sample size was limited. A total of 110 *H. pylori*-infected subjects with no eradication history were included and randomized into VPZ (20 mg b.i.d.) with low- (1000 mg b.i.d.) or high-dose (1000 mg t.i.d.) amoxicillin groups for 14 days. According to PP analysis, the eradication rates of L-VA and H-VA were 94.1% and 95.9%, respectively, which were considered as acceptable results (11), and the difference in the eradication rates between the two groups was not statistically significant.

Furthermore, we found no factors that might influence the efficacy of VA dual therapy, including gender, age, body mass index, education status, cigarette smoking, alcohol drinking, family-based *H. pylori* infection, and indications. Our results showed a high efficacy of 14-day VA dual therapy as a first-line treatment of *H. pylori* infection in China. Gao et al. (23) conducted a real-world retrospective clinical study to analyze the efficacy of 10 mg or 20 mg VPZ twice a day and 3000 mg amoxicillin per day for 14 days as a rescue treatment of *H. pylori* infection. The results revealed a 92.5% eradication rate for VA dual therapy in subjects with previous treatment failure. Inconsistent with our results, Chey et al. (24) reported that 14-day VA dual therapy (20 mg VPZ b.i.d. and 1000 md amoxicillin t.i.d.) only achieved a 77.2% eradication rate in the United States and Europe, which is considered as unacceptable and remains unexplained. The amoxicillin resistance rate in the United States and Europe is relatively low (≤5%) (25). The differences in the efficacy of 14-day VA dual therapy reported in different regions might be explained by race, body mass index, or protocol differences.

In our study, only one patient in each group took <80% of the study drug; therefore, the compliance of the L-VA and H-VA groups was good. Only 29.1% of patients in the L-VA group and 20.0% of patients in the H-VA group reported TEAEs (with no serious TEAEs), indicating that 14-day VA dual therapy is associated with low adverse effects and good safety. Our previous study (9) demonstrated that the TEAE rate is 29.7% and 24.3% in patients receiving L-VA or H-LA for 10 days, respectively. Therefore, a prolonged duration of VA dual therapy from 10 days to 14 days might not lead to an increased incidence of TEAEs.

There were some limitations in this study. First, the sample size was limited to 110 patients all from one center. Further centers with larger samples, different amoxicillin resistance rates, and different races are needed. Second, the efficacy of 14-day L-VA and H-VA in *H. pylori*-infected subjects with previous treatment failure was not examined in this study. Third, endoscopies were conducted in a subset of the included subjects. Antibiotic resistance of amoxicillin was not analyzed so we were unable to determine whether the failure of VA dual therapy was due to amoxicillin resistance.

In conclusion, 14-day L-VA and H-VA provide high efficacy, low adverse effects, and good safety as first-line treatments of *H. pylori* infection in China. This supports VA dual therapy as an alternative regimen for *H. pylori* eradication, especially in an era of increasing antibiotic resistance.

## Data availability statement

The original contributions presented in the study are included in the article/supplementary material. Further inquiries can be directed to the corresponding authors.

## Ethics statement

The studies involving human participants were reviewed and approved by Ethics Committee of The First Affiliated Hospital of Nanchang University. The patients/participants provided their written informed consent to participate in this study. Written informed consent was obtained from the individual(s) for the publication of any potentially identifiable images or data included in this article.

## Author contributions

YH, XX, X-SL, and CH participated in recording the basic information and follow-up of patients; Y-BO, N-SL, CX, and CP provided suggestions for the study design and participated in analyzing data; Z-HZ and YX provided suggestions for the study design and revised the manuscript; YH, XS, YZ, DG, and N-HL designed the study and wrote and edited the manuscript. All authors contributed to the article and approved the submitted version.
